# Wisp2 disruption represses Cxcr4 expression and inhibits BMSCs homing to injured liver

**DOI:** 10.18632/oncotarget.22006

**Published:** 2017-10-24

**Authors:** Dan Qin, Yi Yan, Bian Hu, Wanpo Zhang, Hanmin Li, Xiaodong Li, Shenghui Liu, Depeng Dai, Xiongji Hu, Xingxu Huang, Lisheng Zhang

**Affiliations:** ^1^ College of Veterinary Medicine, University of Huazhong Agricultural, Wuhan 430070, People's Republic of China; ^2^ School of Life Science and Technology, Shanghai Tech University, Pudong New Area, Shanghai 201210, People's Republic of China; ^3^ Hepatic Disease Institute, Hubei Provincial Hospital of Traditional Chinese Medicine, Wuhan 430061, People's Republic of China

**Keywords:** rat, Wisp2, Cxcr4, BMSCs, homing

## Abstract

Liver regeneration/repair is a compensatory regrowth following acute liver failure, and bone marrow-derived mesenchyme stem cell (BMSC) transplantation is an effective therapy that promotes liver regeneration/repair. Wnt1 inducible signaling pathway protein 2 (Wisp2) is highly expressed in BMSCs, however, its function remains unclear. In this work, we used clustered regularly interspaced short palindromic repeats (CRISPR)-associated protein -9 nuclease (CRISPR/Cas9) genome editing technology to knockdown Wisp2 in BMSCs, and these modified cells were then transplanted into rats which were induced by the 2-AAF/PH. By linking the expression of Cas9 to green fluorescent protein (GFP), we tracked BMSCs in the rats. Disruption of Wisp2 inhibited the homing of BMSCs to injured liver and aggravated liver damage as indicated by remarkably high levels of ALT and AST. Moreover, the key factor in BMSC transplantation, C-X-C chemokine receptor type 4 (Cxcr4), was down-regulated in the Wisp2 depleted BMSCs and had a lower expression in the livers of the corresponding rats. By tracing the GFP marker, more BMSCs were observed to differentiate into CD31 positive endothelial cells in the functional Wisp2 cells but less in the *Wisp2* gene disrupted cells. In summary, Wisp2 promotes the homing of BMSCs through Cxcr4 related signaling during liver repair in rats.

## INTRODUCTION

Liver cancer is one of the most frequently diagnosed cancers worldwide and one of the leading causes of cancer death. An estimated 782,500 new liver cancer cases and 745,500 deaths occurred worldwide in 2012. Half of these cases and deaths were estimated to occur in China [1, 2]. Acute liver injury (ALI) and acute liver failure (ALF) are syndromes characterized by a rapid loss of functional hepatocytes in patients with no evidence of pre-existing liver disease. Despite aggressive medical management, many patients with ALF deteriorate severely, and liver transplantation remains their only option for survival [3]. Because of the limited donor availability, attention has been focused on the possibility of restoring liver mass and function through cell transplantation [4]. Recently, studies have documented that bone marrow cells can migrate to injured liver and differentiate into local cell types in the absence of resident cell proliferation. Thus, bone marrow cells can be considered a pool from which exogenous cells could be derived for liver regeneration/repair [5], and among those cells, bone marrow-derived mesenchyme stem cells (BMSCs) have the most potential [6].

BMSCs can be cultivated from bone marrow aspirates as plastic adherent cells *in vitro* [7]. It is a type of stem cell with powerful proliferative and differential potential that represent an attractive tool for the establishment of successful stem cell-based therapy for liver diseases. A number of different stromal cells contribute to the therapeutic effects exerted by BMSCs because BMSCs can differentiate into functional hepatic cells and produce a series of growth factors and cytokines capable of suppressing inflammatory responses, reducing hepatocyte apoptosis, reversing liver fibrosis and enhancing hepatocyte functionality [8]. Besides, BMSCs have an inherent capacity to home to an injured liver and enhance wound healing [9]. BMSCs are safer than embryonic stem cells to use *in vivo* due to their higher chromosomal stability and lower tendency to form neoplasms in the recipient host [10]. However, many researchers found that these cells are poorly efficient in cell transplantation [11, 12]. Retrospective studies have revealed that the low colonization of transplanted MSCs in the liver was the main factor restricting the efficacy of MSC transplantation [13, 14]. To date, an increasing number of factors have been shown to participate in MSC homing, including cytokines, chemokines, growth factors, bioactive lipids, and adhesion molecules [9]. Despite this general understanding of MSC homing, much work is needed to elucidate the molecular mechanisms responsible for this process.

Wnt1 inducible signaling pathway protein 2 (Wisp2, also known as CCN5) is a member of the connective tissue growth factor/cysteine- rich 61/ nephroblastoma overexpressed (CCN) family [15, 16]. It is a 29-kDa protein that is secreted by adipose precursor cells and fibroblasts and is highly expressed in MSCs [17–19]. Early discoveries have led to further research regarding its roles in cell signaling, proliferation, adhesion, invasion, wound healing, fibrosis, skeletal development, implantation, epithelial-mesenchyme transition, angiogenesis and cancers [20–23]. Wisp2 has both growth-promoting and growth-arresting properties depending on the cell types and the microenvironment of the cells [24]. *Wisp2* mRNA and protein were overexpressed in preneoplastic and cancerous cells in human breast. Moreover, the disruption of Wisp2 signaling in MCF-7 cells using antisense oligomers caused a significant reduction in tumor cell proliferation [25]. However, the loss of Wisp2, which is observed in colorectal cancer (CRC), may lead to increased tumor cell invasion and disease progression. Wisp2 may, thus, has potential for therapeutic strategies for the treatment of CRC metastasis [20]. In addition, secreted Wisp2 is a novel regulator of canonical Wnt activation and maintains MSCs in an undifferentiated state. Simultaneously, Wisp2 inhibited Pparγ and associated adipose genes and, similarly to WNT3a, promoted the partial dedifferentiation of the cells and the induction of a myofibroblast phenotype, leading to the activation of markers of fibrosis [17]. Recently, researchers found that Wisp2 might be an important regulator of BMSCs in the regulation of proliferation and initiation of specific differentiation pathways [18, 24], but little is known regarding its mechanism.

To better understand the mechanism underlying the impact of Wisp2 *in vitro* and *in vivo*, we used clustered regularly interspaced short palindromic repeats (CRISPR)- associated protein-9 nuclease (CRISPR/Cas9) gene editing technology [21] to knockdown Wisp2 expression. One of the most striking findings of this analysis was an apparent down-regulation of C-X-C motif receptor 4 (Cxcr4) both *in vitro* and *in vivo*, followed by the loss of Wisp2. Furthermore, by tracking GFP-labeled BMSCs, we found that the loss of Wisp2 resulted in reduced homing and a deterioration of liver function. Notably, CD31, which is the endothelial cell specific marker, was significantly suppressed in the Wisp2 disrupted BMSC transplanted rats. These results illustrate that Wisp2 controls the homing of BMSCs that maybe related to Cxcr4 signaling during liver repair and thus provide a therapeutic roadmap for achieving hepatic regeneration and repair.

## RESULTS

### High expression of Wisp2 in BMSCs

**Figure 1:**
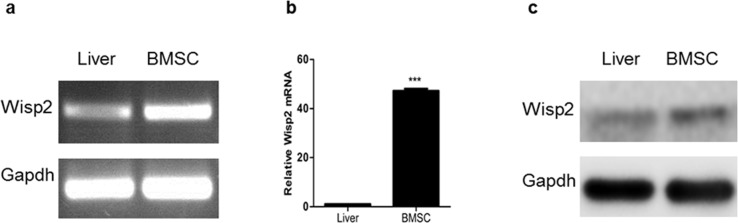
High expression of Wisp2 in BMSCs Total RNA was extracted from rat BMSCs or rat livers, then PCR **(a)** and qRT-PCR **(b)** were performed to examine Wisp2 mRNA level. Error bars represent the ± S.E.M. of triplications. “^***^”, p<0.001. Gene expression level of the samples normalized to Gapdh and the expression of Wisp2 in liver was set as 1. **(c)** The protein level of Wisp2 was measured in BMSCs and livers with western blot analysis. 20μg total protein per lane and Gapdh was used as an internal control.

Wisp2 has been reported to be expressed in BMSCs, to explore the function of Wisp2, we detected its expression by PCR and western blot. As shown in Figure 1a, and 1c, Wisp2 was highly expressed in BMSCs but lowly expressed in rat liver.

### Establishment of Wisp2 knockdown cells by CRISPR/Cas9 system

**Figure 2:**
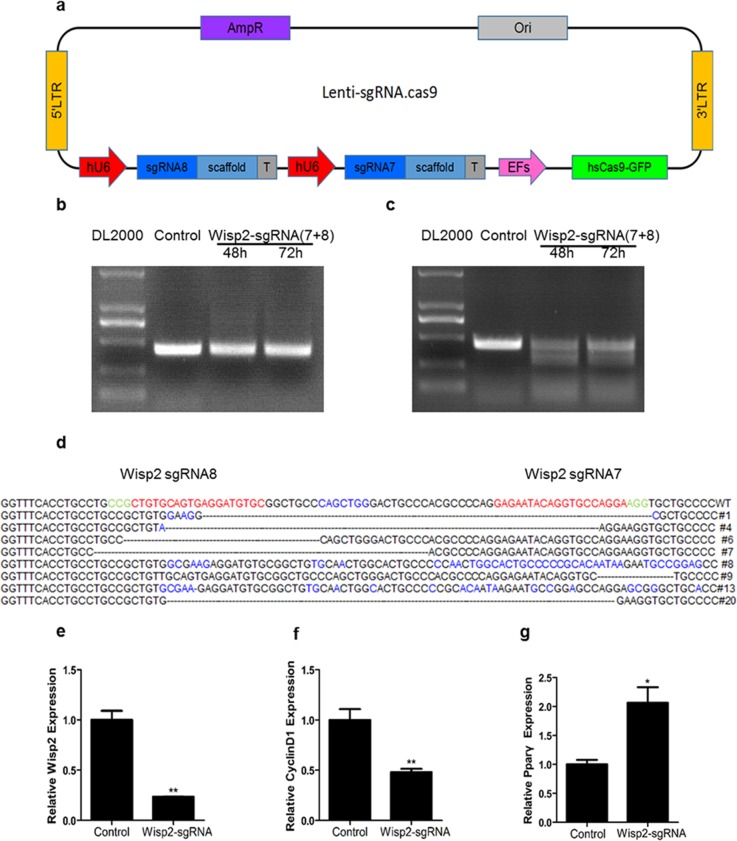
Establishment of Wisp2 knockdown cells by Crispr/Cas9 system **(a)** Sketch of the “all in one” lentivirus expression vector containing Cas9 and sgRNAs (Lenti.sgRNAs.Cas9). SgRNAs insertion sites are highlighted in blue. **(b** and **c)** High cutting effiency of lentivirus against Wisp2 in C3H10T1/2 cells. PCR products were amplified and subjected to T7EN1. Control, C3H10T1/2 infected with basic vector; sgW(7+8), cells infected with lentivirus against Wisp2 containing sgW(7+8); supernatants containing lentivirus were collected 48 hours and 72 hours after the transfection. **(d)** Sequences of modified Wisp2 detected in C3H10T1/2 cells. 8 of 15 TA clones from the PCR products were analyzed by DNA sequencing and displayed in the images. The PAM sequences are highlighted in green; the targeting sequences are highlighted in red; the mutations are highlighted in blue; deletion (-); insertions (^), lower case; ^#^: clone number. **(e-g)** QRT-PCR analysis of the expression of Wisp2, CyclinD1 and Pparγ in C3H10T1/2 cells infected with lentivirus against Wisp2. CyclinD1 and Pparγ were used as positive controls. Gene expression level of the control samples normalized to 36B4 was set as 1. Results are shown as mean ± S.E.M. of 3 independent experiments. “^**^”, p<0.01, “^*^”, P<0.05.

To further investigate the role of Wisp2 in the process of liver repair, the CRISPR/Cas9 system was adopted and eight sgRNAs were designed and worked in pairs (Table 1). The efficiencies of all sgRNAs were first tested by cloning into the Px330 vector and then transfecting into NIH3T3 fibroblasts. It is shown that different sgRNAs had different cutting efficiency. Notably, sgW(5+6) and sgW(7+8) had higher specificity (Supplementary Figure 1a). Next, we cloned these two pairs into a lentivirus vector (Figure 2a) and verified their cleavage efficiency by transducing into Hep1-6 cells. The T7EN1 cleavage bands were visible in the targeted genes (Supplementary Figure 1b and 1c). These data demonstrated that the designed sgRNAs worked effectively with Cas9 in the targeted genes in the cultured cells.

**Table 1:** Sequences of sgRNAs

sgRNAs	Sequences
Wisp2 sgRNA1	GGGTACCCCTGGTGCTGGA
Wisp2 sgRNA2	GTGTCCAAGGACAGGCACA
Wisp2 sgRNA3	GGCCTGGTTTGTCAGCCTG
Wisp2 sgRNA4	CAGACATGCAGGTGGTCGC
Wisp2 sgRNA5	GTGAATGGCCGCAGGTACC
Wisp2 sgRNA6	TCATCCTCTTCGACTGCAC
Wisp2 sgRNA7	GAGAATACAGGTGCCAGGA
Wisp2 sgRNA8	GCACATCCTCACTGCACAG

To obtain stable knockdown cell lines, a lentivirus plasmid containing sgW(7+8) was co-transfection with pMD2.G and PsPAX2 in 293T cell lines for lentivirus generation. Then, the C3H10T1/2 cells, which are murine mesenchymal stem cells, were infected with lentivirus targeting Wisp2 or control virus. For further verification, a T7EN1 cleavage assay was performed, and the cleavage bands were visible in the Wisp2-sgRNA group (Figure 2b and 2c). In addition, the cleavage was characterized by Sanger sequencing, which displayed overlapped peaks in the sequencing chromatographs (Figure 2d). Furthermore, the mRNA expression of *Wisp2* was determined by qRT-PCR. As shown in Figure 2e, Wisp2 expression was significantly decreased in the Wisp2-sgRNA group.

Wisp2 is reported to be correlated positively with Cyclin D1 and inhibits Pparγ activation. Our data showed that disruption of Wisp2 significantly decreased Cyclin D1 abundance (Figure 2f) and increased Pparγ abundance (Figure 2g).

Altogether, these results showed that the selected sgRNAs worked effectively with Cas9 and the CRISPR /Cas9 system functions well *in vitro*.

### The expression of Cxcr4 was reduced in the Wisp2 genetically modified cells

**Figure 3:**
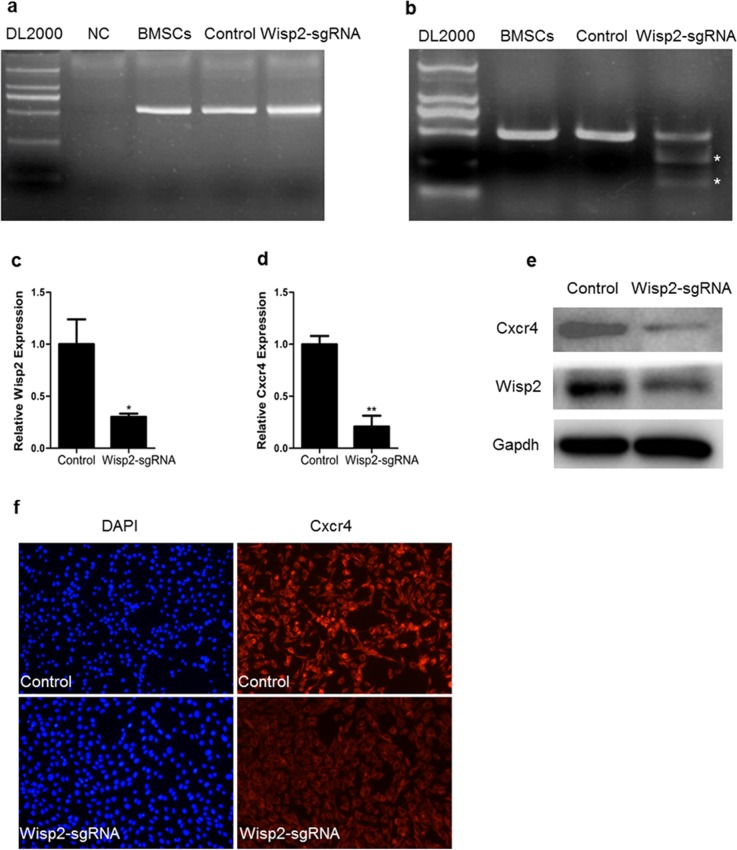
Down-regulation of Cxcr4 in Wisp2 genetically modified cells **(a)** PCR products of the targeted Wisp2 from BMSCs infected with lentivirus against Wisp2 (Wisp2-sgRNA) or lentivirus without sgRNAs (Control). **(b)** Cas9 mediated on-target cleavage of Wisp2 by T7EN1. PCR products were amplified and subjected to T7EN1. Samples with cleavage bands were marked with an asterisk“^*^”. **(c** and **d)** QRT-PCR analysis of the expression of Wisp2 and Cxcr4 in BMSCs infected with lentivirus against Wisp2. The error bars indicated the ± S.E.M. of at least three independent experiments, “^*^”, p<0.05. Gene expression level of the control samples normalized to Gapdh was set as 1. **(e)** Wisp2 and Cxcr4 protein expression levels were depressed in the Wisp2-sgRNA-BMSCs. Total cellular protein was analyzed by immunoblotting for Gapdh. 20μg of total protein per lane. **(f)** Immunofluorescence showed that Cxcr4 was reduced in the Wisp2-sgRNA-BMSCs. Blue, DAPI; Red, Cxcr4.

Based on the experiment above, BMSCs were infected with CRISPR/Cas9 lentivirus targeting the *Wisp2* gene or control virus, and Supplementary Figure 2a represented high-efficiency retroviral infection of BMSCs. Subsequently, genomic DNA was extracted and PCR and T7EN1 digestion were performed, the T7EN1 cleavage bands were visible in the targeted gene (Figure 3a and 3b). At the molecular level, the cleavage was characterized further by Sanger sequencing, which displayed overlapped peaks in the sequencing chromatographs (Supplementary Figure 2b). Next, a routine determination was performed via qRT-PCR to detect Wisp2 expression. Unsurprisingly, the mRNA level of *Wisp2* was significantly reduced (Figure 3c).

According to previous studies, Cxcr4 stimulates the migration of MSCs *in vitro* and is known to be required for the migration of MSCs *in vivo*, particularly in the liver. Thus, the expression of Cxcr4 in the Wisp2-sgRNA cells was analyzed, and the abundance of Cxcr4 was shown to be dramatically decreased (Figure 3d). Then, Cxcr4 was examined by immunoblotting and immunostaining. As shown in Figure 3e and Figure 3f, Cxcr4 was markedly reduced in the Wisp2-sgRNA BMSCs. The same phenomenon was also detected in the C3H10T1/2 cells at the mRNA level (Supplementary Figure 2c). Besides, the mRNA expression of *Cxcr7* was reduced, concomitant with the decreased *Cxcr4* in both modified cell lines (Supplementary Figure 2d and 2e).

Altogether, the loss of Wisp2 significantly reduced the expression of Cxcr4 in the cultured cells from both mice and rats.

### Impaired liver repair in the lentiviral Wisp2 disrupted BMSC transplantation rats after 2-AAF/PH induced liver injury

**Figure 4:**
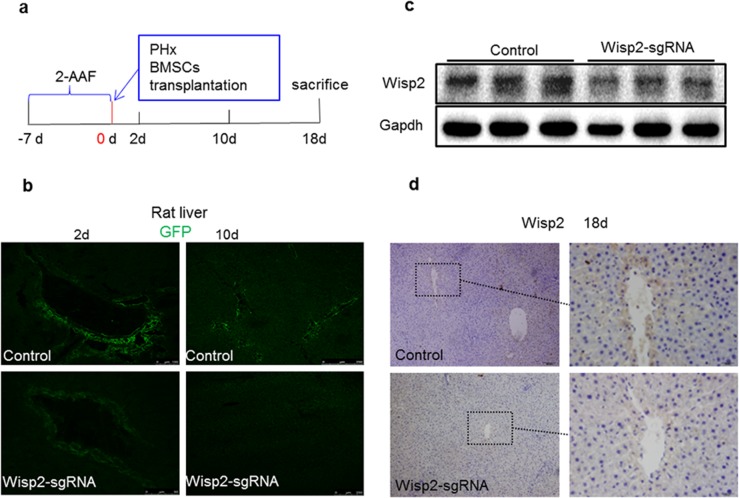
Wisp2 was disrupted in rat livers after 2-AAF/PH **(a)** Seven days after the intragastric administration of 2-AAF, the rats underwent PH and were transplanted with 1.8×10^6^ Wisp2-sgRNA-BMSCs or equal amounts of control BMSCs and then sacrificed and livers collected as indicated in the scheme. **(b)** Representative images showing homing BMSCs to the injured liver. **(c** and **d)** Wisp2 expressed in rat livers. Expression of Wisp2 was detected in rat livers 18 days post-transplantation by Immunoblot analysis (c). 40μg of total protein per lane. (d) Immunostaining was performed. Original magnification: 10× (Left panel) and 40× (right panel).

To further determine the contribution of Wisp2 to BMSC-mediated liver regeneration and repair, the Wisp2 modified BMSCs were transplanted into injured livers of rats subjected to 2-AAF/PH (Figure 4a). By tracing GFP, BMSCs were detected in rat liver at day 2 and day10 in Figure 4b. Compared to the control group, the expression of Wisp2 was significantly decreased in the Wisp2-sgRNA group (Figure 4c and 4d).

**Figure 5:**
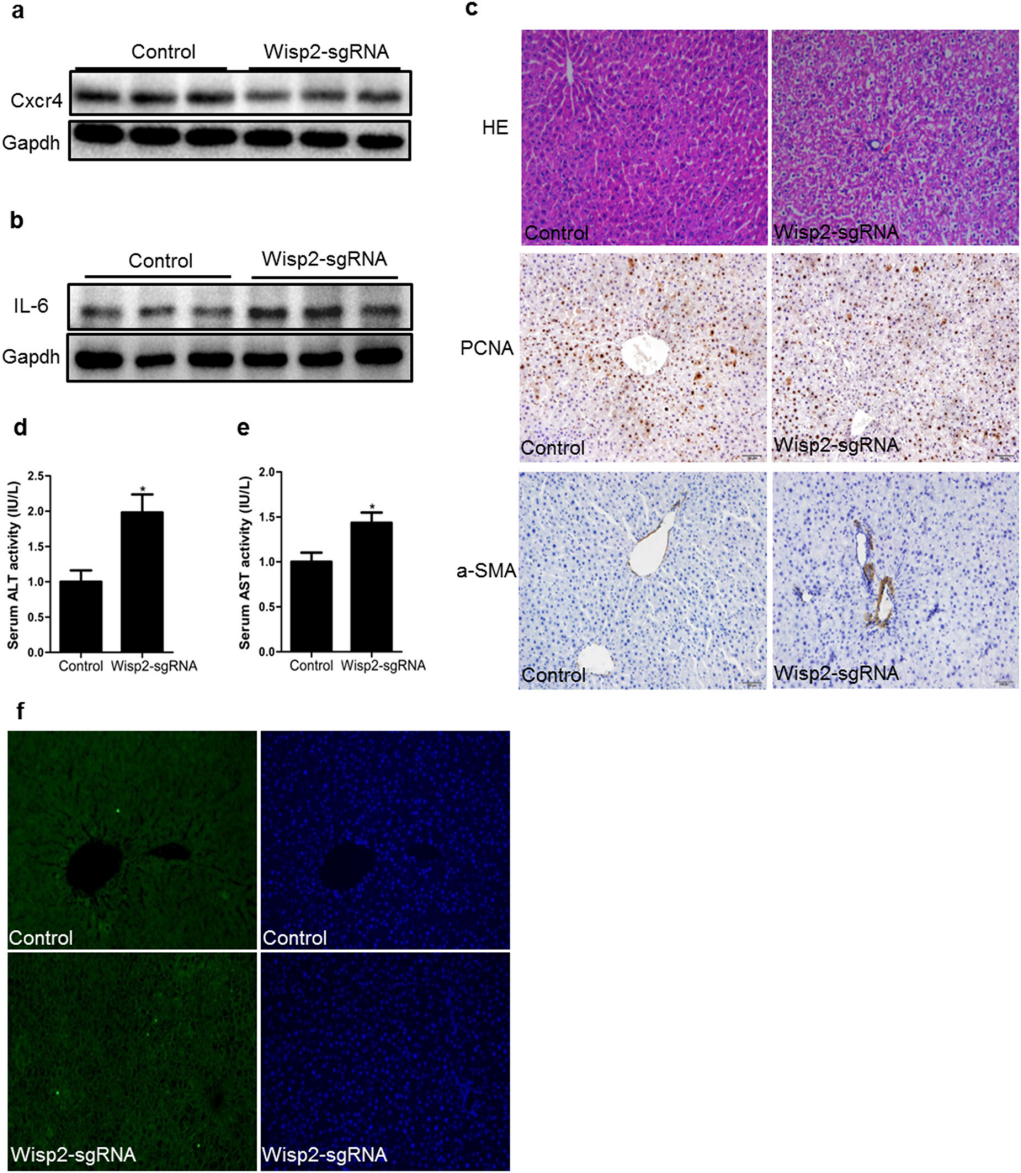
Cxcr4 was decreased and repair capability was reduced in lentiviral Wisp2 disrupted BMSC transplantation rats Liver samples treated with Wisp2-sgRNA or not were examined for the expression of Cxcr4 **(a)** and IL-6 **(b)**. 40μg of total protein per lane, as indicated by western analysis 18 days after transplantation. **(c)** Paraffin- embedded sections from livers obtained 18 days after the transplantation were stained with H&E and PCNA or α-SMA. The Wisp2-sgRNA group showed more extensive liver damage and cellular swelling (20×). Serum ALT **(d)** and AST **(e)** levels were assessed to determine the degree of liver injury. The control group was set as 1. Asterisks indicate significant differences, “^*^”, P< 0.05. **(f)** Representative micrographs of TUNEL staining of liver tissue. Liver sections collected 18 days after BMSCs transplantation were stained with FITC-conjugated TUNEL to identify apoptotic cells. The results showed that disrupted Wisp2 incresed the levels of apoptosis caused by 2-AAF/PH. Original magnification: 20×.

Our preceding results indicated that the loss of Wisp2 disrupted the expression of Cxcr4 from both mice and rat MSCs, we, therefore, investigated whether Wisp2 played the same role in this liver repair model. As expected, Cxcr4 was reduced in the Wisp2 deletion group compared to that in the functional Wisp2 BMSC transplanted group (Figure 5a). Additionally, the pro-inflammation factor, interleukin-6 (IL-6) was increased in the Wisp2 disrupted group (Figure 5b).

To investigate the liver histology of the rats after transplantation of the BMSCs, hematoxylin and eosin (H&E) staining was performed. Compared to the control group, the livers in the Wisp2-sgRNA group exhibited cell shrinkage with a disappeared cytoplasm and vacuolar degeneration (Figure 5c). Additionally, some of the hepatocytes dissolved, allowing the cells to develop soluble necrosis. Furthermore, some of these cells disintegrated directly, and cellular debris could be observed (Supplementary Figure 3a). Proliferating cell nuclear antigen (PCNA) and α-smooth muscle actin (α-SMA) staining were based on H&E staining to detect the proliferation and vascular fibrosis. PCNA staining revealed that growth efficiency was much lower in Wisp2 gene disruption group, the result of α-SMA staining was exactly the opposite (Figure 5c).

Since ALT and AST are commonly measured clinically as a part of the diagnostic evaluation of hepatocellular injury, we compared the serum ALT/AST in the control rats and the Wisp2-sgRNA BMSC transplanted rats. Predictably, the serum AST and ALT levels were significantly higher in the Wisp2 depleted rats (Figure 5d and 5e). Similarly, terminal deoxynucleotidyl transferase 2-deoxyuridine, 5-triphosphate nick end labeling (TUNEL) staining revealed that disrupted Wisp2 induced apoptosis compared with the control group (Figure 5f). Thus, we demonstrated that loss of Wisp2 prevented the damaged liver from repairing.

### Deletion of Wisp2 reduced the *in vivo* homing of transplanted BMSCs to the injured livers related to Cxcr4 signaling

**Figure 6:**
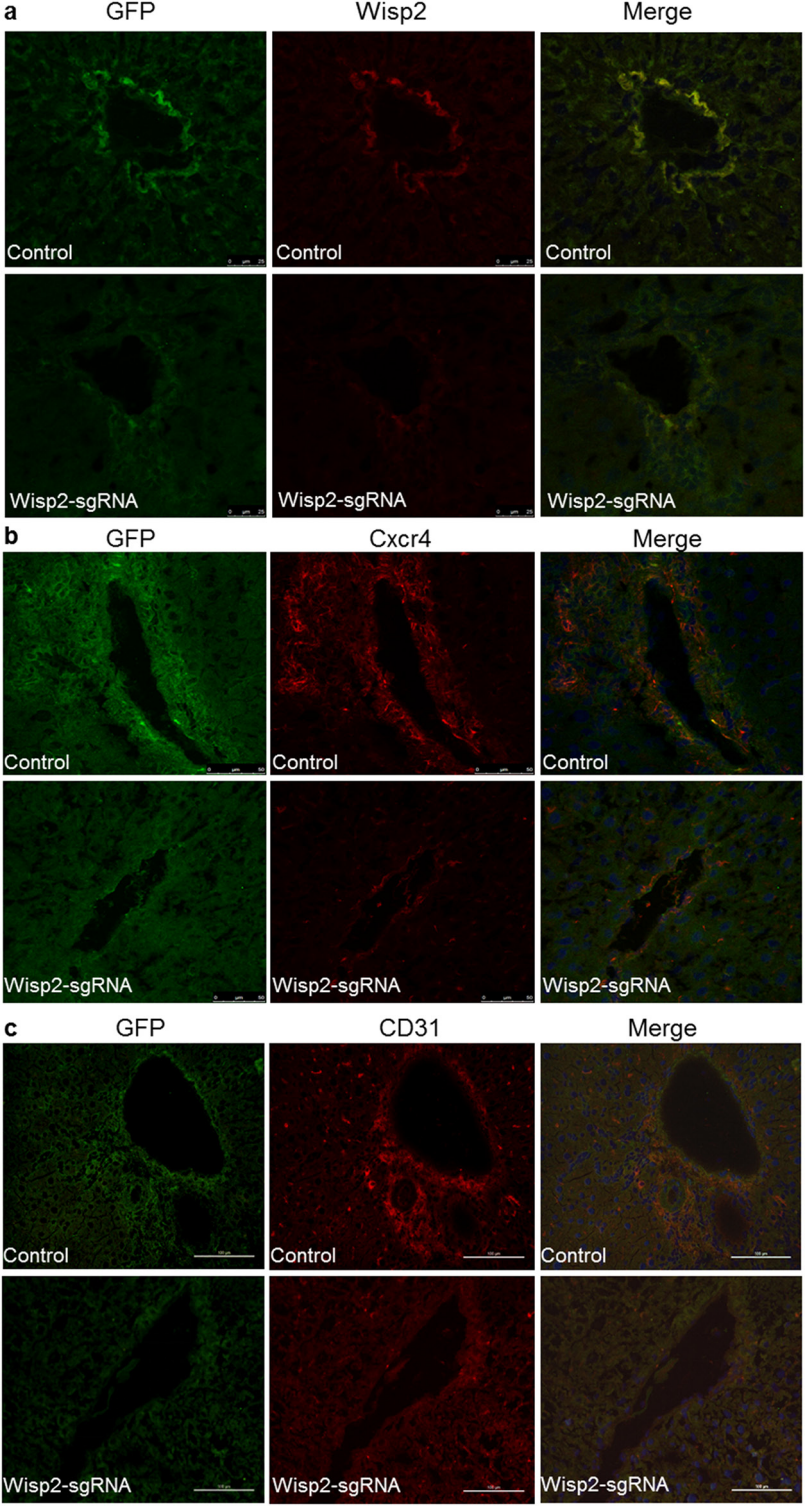
Wisp2 was required for BMSC migration and differentiation to endothelial cells Significantly decreased expression of Wisp2 and Cxcr4 in rat livers after the Wisp2-sgRNA-BMSC transplanted 18 days, the expression of Wisp2 **(a)** and Cxcr4 **(b)** were determined in liver sections. Liver cyrosections were stained for GFP (Green fluorescence) and Wisp2/Cxcr4 (Red fluorescence). Images were merged with DAPI (Blue fluorescence) staining reveal the nuclei. **(c)** Expression of the endothelial cell marker CD31 (Red fluorescence) was also examined in the liver sections by immunostaining. The expression of CD31 was reduced in the Wisp2-sgRNA BMSCs transplanted rat livers.

To elucidate the mechanism underlying the pro-regeneration and repair of BMSCs niche, gene expression was detected by immunofluorescence staining. The expression of Wisp2 was conditionally ablated in the Wisp2-sgRNA group (Figure 6a). Figure 6b indicated that Cxcr4 was reduced in the Wisp2 deletion group compared to that in the functional Wisp2 BMSC transplanted group. In addition, by tracing GFP, we found that the homing efficiency of the functional Wisp2 BMSCs in the transplanted rats was much higher than that in the Wisp2 modified rats (Figure 6a-6c). Meanwhile, the transplanted BMSCs spread from the blood vessels along the hepatic sinus to the periphery (Supplementary Figure 3b).

To determine how BMSCs modulated liver regeneration and repair, specific markers were detected, including CD31, cytokeratin 19(CK19) and von willebrand factor(vWF). Finally, we discovered that CD31 was much more highly expressed in control group than in the Wisp2-sgRNA group (Figure 6c). Compared to CD31, CK19 staining displayed less co-expression and more self-signal (Supplementary Figure 4a), in contrast, hardly positive vWF co-dyeing signal could be observed (Supplementary Figure 4b).

Hence, it was possible that BMSCs repair liver injury by endothelial cell proliferation and angiogenesis through Wisp2 mediated Cxcr4 signaling.

## DISCUSSION

Recently, the possibility of using organ- or tissue- specific adult stem cells has been examined in both fields of gene and cell transplantation therapy [5]. One of the main attractions was MSCs, particularly for the treatment of ALF [13, 22]. Unfortunately, many researchers have found that cell engraftments have a poor efficacy [23]. This report showed that Wisp2 knockdown by CRISPR/Cas9 system inhibited BMSCs homing to injured livers through Cxcr4 related signaling and delayed liver repair.

MSCs exhibit multiple beneficial properties due to their capacity for homing, attenuating the inflammatory response, modulating immune cells, and promoting tissue healing [26]. The great benefit of BMSCs is that they are easily accessible through aspiration of the patient's bone marrow, so that the use of BMSCs avoids ethical issues, facilitating their application both for auto- and allo-transplantation. BMSCs are also easily expanded on a large scale, which is very convenient for clinical use [27]. Here, we transplanted BMSCs labeled with GFP via the tail vein into rats that underwent liver injury by a 2-AAF/PH treatment. GFP was used as a reporter to detect the transplanted BMSCs. Two days later, BMSCs were found around the blood vessels, and their abundance increased at day 10. This finding is consistent with previous reports [10, 28].

The most novel observation in this report is the delayed liver repair in the Wisp2-sgRNA rats compared to the control rats after the liver injury. These results identified an unexpected role of Wisp2 directed BMSCs in regulating liver repair. Wisp2 has been reported to be involved in cell proliferation, migration, wound healing and angiogenesis [29]. Subsequently, a study has demonstrated that the inhibition of Wisp2 at the mRNA and protein levels can effectively suppress the proliferation of cells [30]. These findings are consistent with our results, as we found that more PCNA staining signal in control group, in contrast, IL-6 and α-SMA were increased in Wisp2-sgRNA group. We further showed that the expression of Cxcr4 was largely decreased in the Wisp2-sgRNA rats and the Wisp2-sgRNA cells. Intriguingly, BMSCs express relatively high levels of Wisp2 and Cxcr4 [31].

Cxcr4, which is a G-protein-coupled receptor, is highly important due to its critical role in development regulation, hematopoiesis, angiogenesis, stem cell migration and wound healing [32]. Ding has demonstrated that after an acute liver injury, Cxcr7 up-regulation in liver sinusoidal endothelial cells acts in conjunction with Cxcr4 to induce transcription factor Id1, which deploys pro-regenerative angiogenic factors and triggers regeneration. This phenomenon was abrogated by genetic silencing of either Cxcr4 or Cxcr7 [28]. Dmitriev has proposed that Dux4 controlled the cellular migration of MSCs through the Cxcr4 receptor. Adding Cxcr4 antibody to the culture medium completely abolished the effect of Dux4 overexpression on cell migration [32]. In addition, it has been reported that the overexpression of Cxcr4 improved the efficiency of infused MSCs homing toward the damaged tissues [33]. Besides, many studies have demonstrated that Cxcr4 played a pivotal role in mobilizing BMSCs to the damaged tissues and promoting wound healing [28, 32, 34–36]. Consequently, the regulation of Cxcr4 expression was expected to influence the directional homing of infused BMSCs. Thus, we proposed that Wisp2 malfunction causing Cxcr4 downregulation might be the major mechanism for delayed liver repair. However, we would like to mention that another study implicated that the constitutive activation of Cxcr4 in LSECs by chronic injury established a pro-fibrotic vascular niche, activating adjacent myofibroblast cells and provoking fibrogenesis [28].Therefore, a more comprehensive investigation of Cxcr4 is needed in the future.

Additionally, the differentiation of BMSCs *in vivo* is also a matter of concern. Studies investigating BMSCs have shown their capability to differentiate into many specific cell types [37–39]. CD31, also named PECAM-1, was a specific marker of endothelial cells [40]. Several studies have suggested that CD31 may be involved in the process of angiogenesis. Studies of human microvascular endothelial cells grown in a three-dimensional culture in the presence of factors that promote angiogenesis support this concept. In these studies, the exposure to anti-CD31 antibodies or soluble CD31 protein was shown to inhibit the formation of normal multicellular tubes [41]. In this study, we noticed that while Wisp2 was knocked down, the reduction of Cxcr4 inhibited the homing of BMSCs towards to the injured liver and simultaneously decreased the number of CD31^+^ endothelial cells by immunofluorescence. The result is consistent with previous reports which showed that BMSCs could be recruited at the sites of neovascularization through the paracrine regulation of their angiogenic properties [42]. Furthermore, we found that BMSCs could differentiate into CK19^+^ cell, but hardly into vWF^+^ cells. Thus, we suggest that knocking down Wisp2 repressed endothelial cell proliferation and angiogenesis and then delayed liver repair.

## MATERIALS AND METHODS

### Ethics statement

Investigation has been conducted in accordance with the ethical standards and according to national and international guidelines and has been approved by Huazhong Agriculture University for the care and use of laboratory animals.

### Cell culture

NIH3T3, Hep1-6 and 293T cells were cultured with 10% fetal bovine serum (FBS, Thermo Fisher, Waltham, USA) in H-DMEM (Thermo Fisher). C3H10T1/2 cells were cultured with 10% FBS in EBSS-MEM (Thermo Fisher) including 1% NEAA (Solarbio, beijing, USA). Rat BMSCs (RASMX-01201) were purchased from Cyagen Biosciences and cultured in DMEM supplemented with 10% fetal bovine serum, 100 IU/mL penicillin-streptomycin, and 2mM glutamine. Cells were all incubated at 37°C in a humidified incubator containing 5% CO_2_.

### Animal model

SD rats, 5-6 weeks of age (170-180 g), were purchased from Hubei Provincial Center for Disease Control and Prevention. These animals were bred in-house and maintained on standard laboratory chow and a daily 12-hour light/12-hour dark cycle. Each rat was given 20 mg/kg 2-Acetylaminofluorene(2-AAF, Sigma Chemicals, CA, USA) dissolved in PEG400 (Servicebio, Wuhan, China) via an intragastric administration once a day for 1 week, and then, two-thirds of their livers were removed [43]. Then, the rats were immediately divided into two groups and underwent transplantation with 1.8×10^6^ Wisp2-sgRNA BMSCs or control BMSCs in a total volume of 500μl by tail vein injection.

### Cas9/ sgRNA efficiency test in cells

The sgRNAs were designed online as shown in Table 1 and cloned into Px330 or a lentiviral plasmid. The transfection procedure was carried out using lipofectamine 2000 reagent (Invitrogen, Carlsbad, USA) according to the manufacturer's instructions. Briefly, the cells were transfected with Px330 containing the sgWisp2 plasmid or basic plasmid by lipofectamine 2000 in a 6-well culture plate. Then, 48 hours after the transfection, genomic DNA was extracted from the cells with a lysis buffer. Next, the DNA was precipitated with alcohol and sodium acetate. Subsequently, a T7EN1 (NEB, New England Biolabs, USA) cleavage assay was performed as described by Shen et al [44]. Briefly, the targeted fragments from the genomic DNA were amplified by a 2×Taq PCR Mix (Ald lab, Beijing, China) and purified using a TYIAN Quick Midi Purification Kit (Tiangen, Beijing, China). The primers used to amplify the Wisp2 targeted fragments are listed in Table 2. The purified PCR products were denatured and re-annealed in NE Buffer 2 (NEB) using a thermocycler (Long Gene, A200, China). The PCR products were digested with T7EN1 for 30 min at 37°C and then separated on a 1.5% agarose gel. The PCR products with mutations detected by the T7EN1 cleavage assay were then sub-cloned into a PMD-18T vector (Takara, Japan). The colonies were randomly selected and sequenced with specific primers listed in Table 2.

**Table 2:** Primers for PCR and qRT-PCR

Genes	primers (5’-3’)	Accession number
r-*Wisp2*	TGCCTGCTCGGGAAGTACGCTTGATTTGGGTGTTTA	NM_031590.1
r-*Cxcr4*	GCATCGTCATCCTGTCCTGTACGCTCTCGAACTCACATCC	NM_022205.3
r-Wisp2-test	TCCTGGTCCTCTTTCTCCAAGGTAGGGCTGGTTATG	NC_005102.4
r-*Cxcr7*	CAGCACCTCCAGCTATAAGAATGGCGAGCAGGAAGTAGA	NM_053352.1
m-*Cxcr4*	AGCATGACGGACAAGTACCGATGATATGGACAGCCTTACAC	NM_009911.3
m-*Wisp2*	TGACAGACGCTCCTGATCTCCACAGCAAGAAAGACCTCCATCCC	NM_016873.2
m-Wisp2-test	GGAGAAACCGAGGCAAGATGTGAGCACTCACTCACCTTGGGCTGA	NC_000068.7
m-*Cyclin D1*	TCAAGTGTGACCCGGACTGATGTCCACATCTCGCACGTC	NM_007631.2
m-*Pparγ*	TGGGTGAAACTCTGGGAGATTCAGAGGTCCACAGAGCTGATTCC	NM_001127330.2
m-*Cxcr7*	GCGTATCAAAGCCAGCACCCAACAGCCTTACACCTCCC	NM_001271607.1
m-*36B4*	TGGAGACAAGGTGGGAGCCCACAGACAATGCCAGGACGC	NM_007475.5

### Lentivirus generation and cell infection

The resulting shuttle vectors with or without sgW(7+8) were mixed with a packaging mix (pMD2.G and PsPAX2). Then, the mixed vectors were added to the opti-MEM (Invitrogen, Carlsbad, USA) culture medium and transfected into the 293T packaging cell line. Recombinant lentivirus expressing both GFP and sgRNAs of Wisp2 were harvested and filtered through a 0.45μm filter (Millipore, USA) after 48 hours or 72 hours. For the infection, the cells were transduced with lentiviruses in 5% FBS growth media supplemented with polybrene (8μg/ml). The cells were incubated overnight with the lentivirus and cultured in fresh 5% FBS growth media for an additional 3 days. Subsequently, the cells were cultured in 10% FBS growth media. Finally, we detected the cutting efficiency as described above.

### Real-time quantitative PCR

Total RNA was isolated and purified using RNAiso plus (Takara, Japan) and transcribed into cDNA using the first strand cDNA synthesis kit (TOYOBO, Japan). The cDNA was diluted for the real-time quantitative PCR (qRT-PCR), and the samples were run in a 10μl reaction system using the SYBR GREEN qPCR mix (TOYOBO, Japan). The data were detected using the ABI CFX Connect TM Real-Time PCR Detection System (ABI, USA). The level of mRNA in each sample was normalized with respect to 36B4 as the internal standard.

### Western blotting

The cells were washed with cold PBS, lysed (Beyotime, Shanghai, China) on ice for a few minutes, and centrifuged at 12,000 g for 5 min at 4°C, and the supernatants containing the total proteins were collected. The supernatants were subjected to SDS-PAGE and immunoblotting. In total, proteins were separated on a 15% polyacrylamide gel, followed by transferring onto a PVDF membrane (Millipore, Billerica, MA, USA). The membrane was blocked for 2 hours with 5% skim milk (Bio sharp, China) and incubated overnight with the anti-Wisp2 (Bioss, bs-5100s), anti-Cxcr4 (Santa Cruz, sc-53534) and anti-Gapdh (Santa Cruz, sc-293335) antibodies. After 3 washes, the secondary antibody was added at a 1:5,000 dilution and incubated at room temperature for 1.5 hours. After 4 washes, the membrane was exposed using western bright TM ECL (Juneng, K-12045-D10) in the imaging system (SYNGENE, G: Box). The protein amount was normalized to the amount of Gapdh as the internal control.

### Serum transaminase levels and histological analysis

The serum aspartate aminotransferase (AST) and alanine aminotransferase (ALT) levels in the rat blood were measured with assays purchased from Nanjing Jiancheng (C010-2, C009-2). For the histologic assessment, the livers were fixed in 4% formaldehyde for 24 hours and embedded in paraffin. Liver sections (4μm) were deparaffinized and fixed. The sections were stained with H&E.

### Immunostaining and histological analysis of liver cryosections

To collect tissues for the histological analysis, the rat livers were snap-frozen in OCT. For the immunofluorescence microscopy, the liver sections (6μm) were blocked (10% goat serum/1% Triton X-100) and incubated in the following primary antibodies: anti-Wisp2 (10μg/ml, Santa Cruz, sc-514070), anti-Cxcr4, anti-GFP (10μg/ml, Proteintech, 50430-2-AP), anti-CD31 (10μg/ml, Santa Cruz, sc-376764), anti-PCNA (10μg/ml, Santa Cruz, sc-25280), anti-α-SMA (10μg /ml, Servicebio, GB13044), anti-CK19 (10μg/ml, Santa Cruz, sc-376126) and anti-vWF (10μg/ml, Santa Cruz, sc-53466). After incubation in fluorophore-conjugated secondary antibodies (2.5μg/ml, Invitrogen, A-11034, A-21424), the sections were counterstained with DAPI (Invitrogen). Finally, confocal microscope and fluorescent microscope were used to observe the results.

### TUNEL staining

In Situ Cell Death Detection Kit, Fluorescein (cat no.11684795510) was purchased from Roche and terminal deoxynucleotidyl transferase 2-deoxyuridine, 5-triphosphate nick end labeling (TUNEL) staining was performed with the manufacturer's instructions.

### Statistical analysis

All data are presented as the mean ± S.E.M. of at least three separate experiments. Differences between groups were tested for statistical significance using Student's two-tailed *t*-tests. Statistical significance was set at P<0.05 (^*^), P<0.01 (^**^).

## CONCLUSIONS

Although much remains unknown, our results confirm a critical role of Wisp2 in promoting liver repair. Moreover, our studies demonstrate that knockdown of Wisp2 repressed Cxcr4 expression and decelerated liver repair. Therefore, both Wisp2 and Cxcr4 participate in the promotion of liver repair.

## SUPPLEMENTARY MATERIALS FIGURES



## Abbreviations

2-AAF: 2-Acetylaminofluorene
α-SMA: α-smooth muscle actin
All in one: One plasmid which contains both GFP, Cas9 and sgRNAs
ALI: Acute liver injury
ALF: Acute liver failure
ALT: Alanine transaminase
AST: Aspartate amino transferase
BMSC: Bone marrow-derived mesenchyme stem cell
CK19: Cytokeratin 19
CRC: Colorectal cancer
CRISPR/Cas9: Clustered regularly interspaced short palindromic repeats (CRISPR)-associated protein -9 nuclease
Cxcr4: C-X-C chemokine receptor type 4
GFP: Green fluorescence protein
IL-6: Interleukin-6
MSCs: Mesenchyme stem cells
PCNA: Proliferating cell nuclear antigen
TUNEL: Terminal deoxynucleotidyl transferase 2-deoxyuridine, 5-triphosphate nick end labeling
vWF: von Willebrand factor
Wisp2: Wnt-1 inducible signaling pathway protein 2.
