# Supplementary material for: Wisp2 disruption represses Cxcr4 expression and inhibits BMSCs homing to injured liver
Source: Oncotarget. 2017 Oct 24;8(58):98823–36. doi: 10.18632/oncotarget.22006 (PMC5716770; doi:10.18632/oncotarget.22006)
Supplement: Supplementary file 1 [file oncotarget-08-98823-s001.pdf]

Wisp2 disruption represses Cxcr4 expression and inhibits BMSCs homing to injured liver

SUPPLEMENTARY MATERIALS

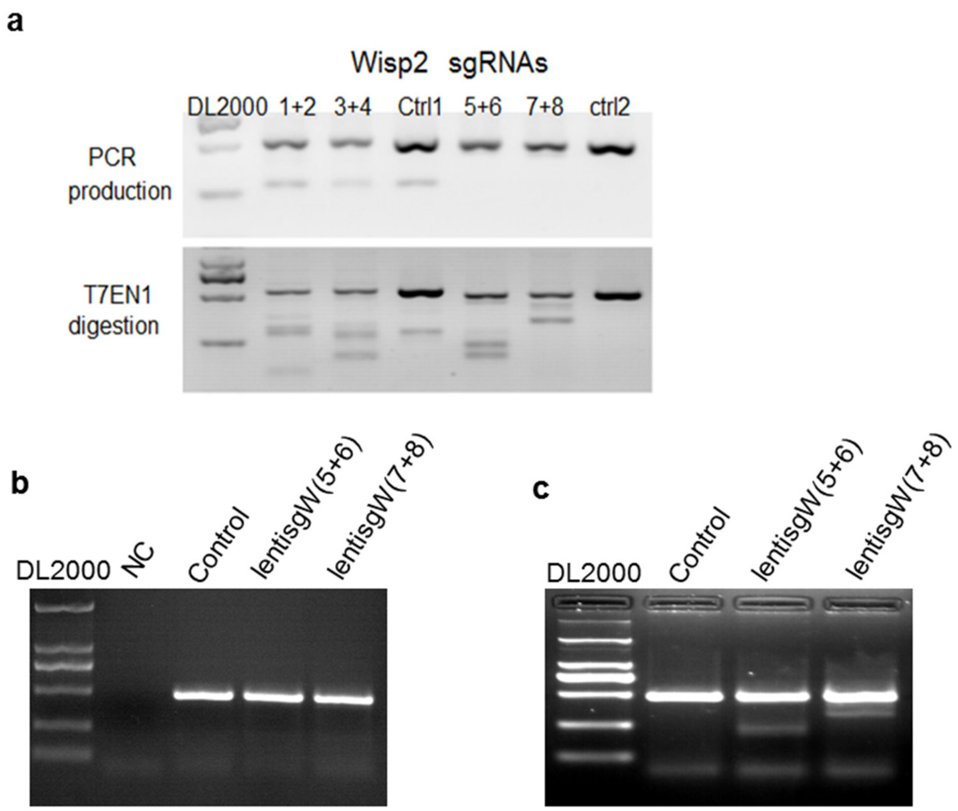

Supplementary Figure 1: sgRNAs: Cas9-mediated modification of Wisp2. (a) PCR products of targeted Wisp2 from NIH3T3 cells transfected with Px330 containing Wisp2 sgRNAs in pairs (top panel). Cas9 mediated on-target cleavage of Wisp2 by T7EN1 (bottom panel). PCR products were subjected to T7EN1. (1+2), Wisp2 sgRNA1 and sgRNA2 worked in pairs and so on; Ctrl1 and Ctrl2, control. **(b** and **c)** Construction of lentivirus vectors carrying W (5+6) or W (7+8) and the detection of the cutting effects of these vectors in hep1-6 cells. The last two groups were inserted into a basic lentivirus plasmid and transfected into Hep1-6 cells for 48 hours. Then, DNA was extracted to detect the cutting efficiency by T7EN1 digestion.

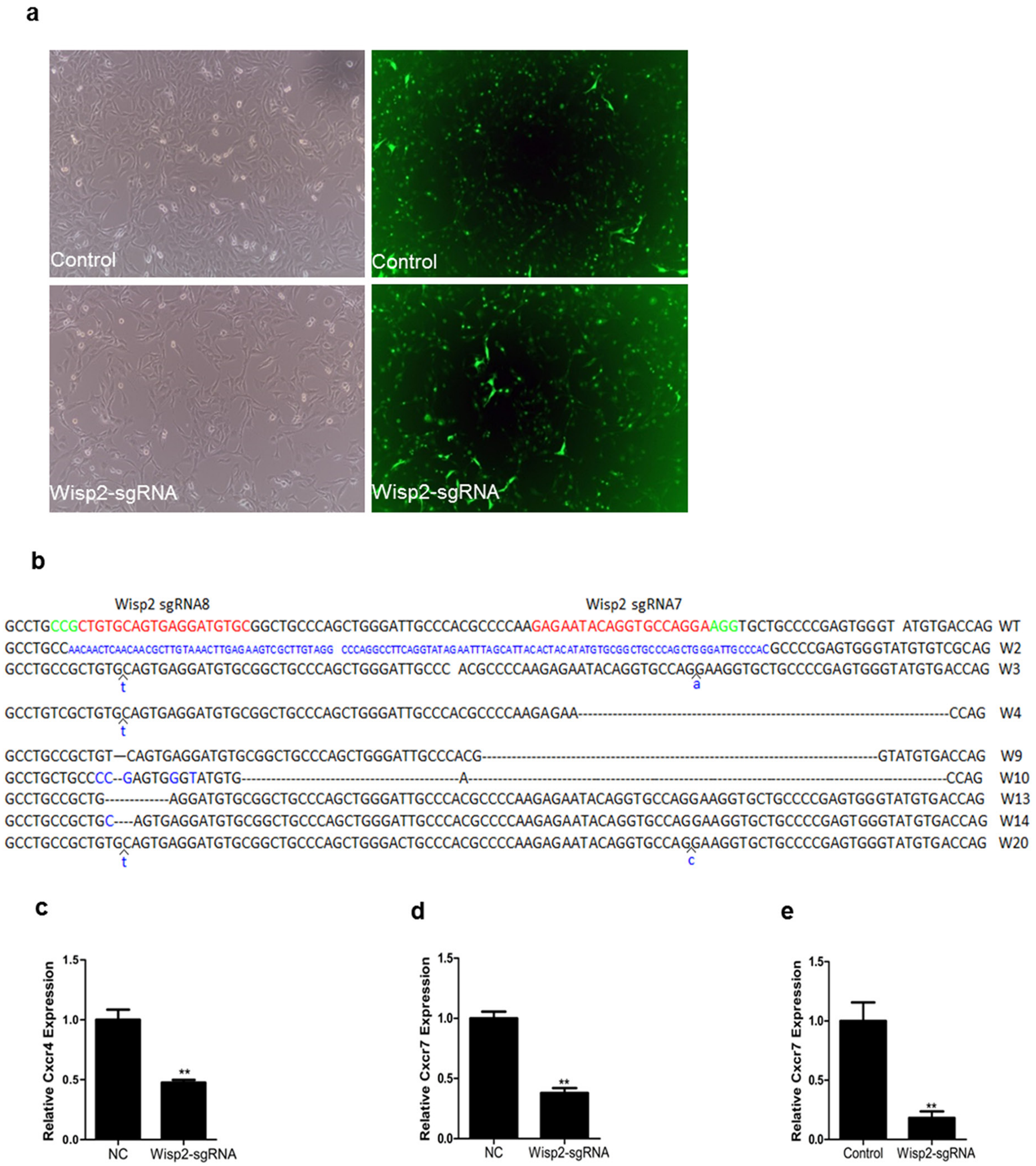

Supplementary Figure 2: Dual sgRNAs for CRISPR/Cas9 targeting on Wisp2 in BMSCs. **(a)** BMSCs were infected with Wisp2-sgRNA or control virus. Left panel, white light sight; right panel, green fluorescence. **(b)** Sequences of modified Wisp2 detected in BMSCs samples. 8 of 15 TA clones from the PCR products were analyzed by DNA sequencing and results showed in the picture. The PAM sequences are highlighted in green; the targeting sequences are highlighted in red; the mutations are highlighted in blue; deletion (-); insertions (^), lower case; W: clone number. **(c)** QRT-PCR analysis of the expression of Cxcr4 in C3H10T1/2 cells infected with lentivirus against Wisp2. **(d** and **e)** QRT-PCR analysis of the expression of Cxcr7 in C3H10T1/2 cells and BMSCs infected with lentivirus against Wisp2.

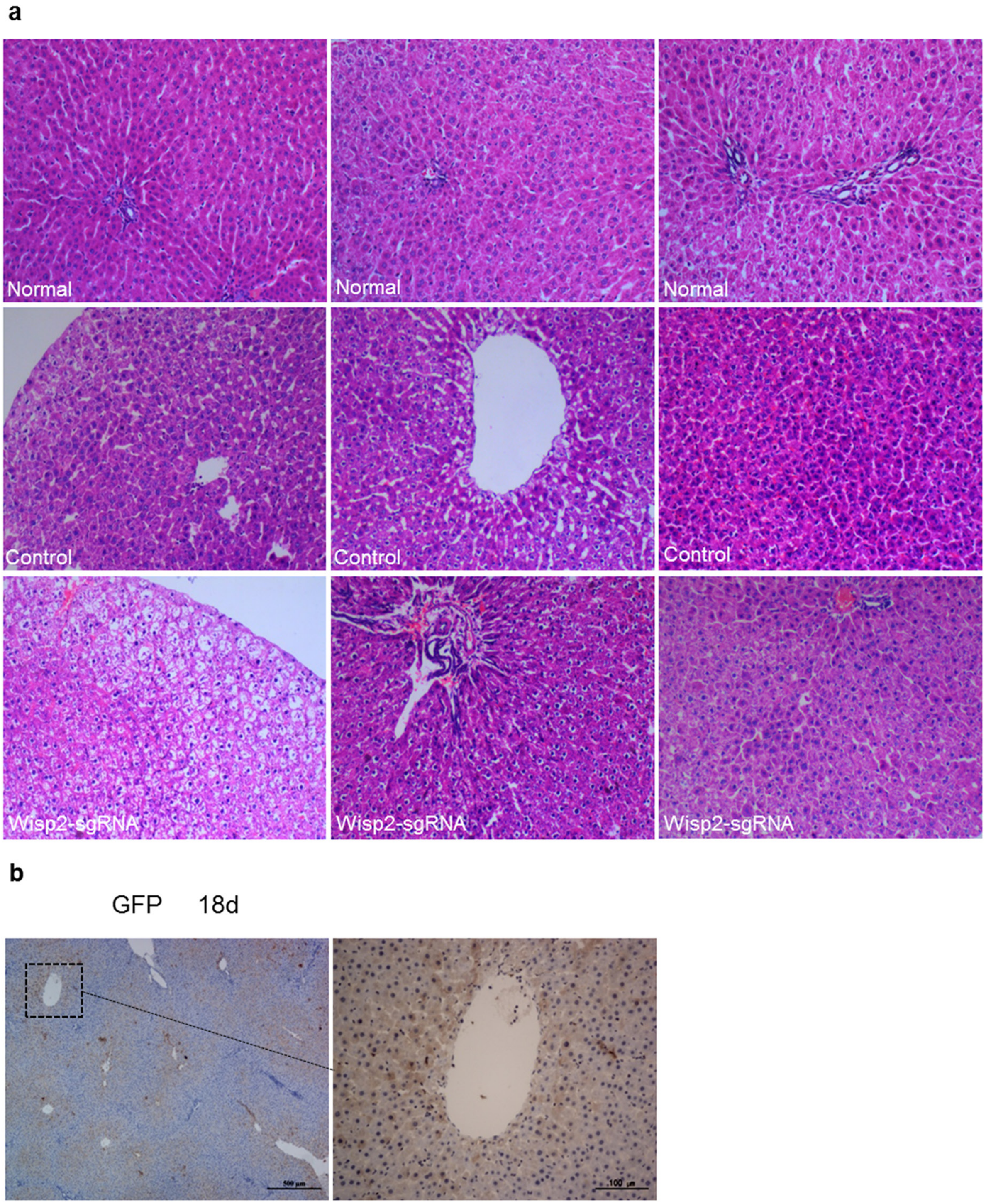

Supplementary Figure 3: Wisp2 knockdown resulted in poor repair capability. Paraffin-embedded sections from livers obtained 18 days after BMSCs transplantation were stained with H&E **(a)** and GFP **(b)**. Normal, normal rat liver; Control, livers of rat transplanted with functional Wisp2 BMSCs; Wisp2-sgRNA, liver of rat transplanted with BMSCs which transfected by lentivirus against Wisp2. Original magnifications: 20×, 10×, 40×.

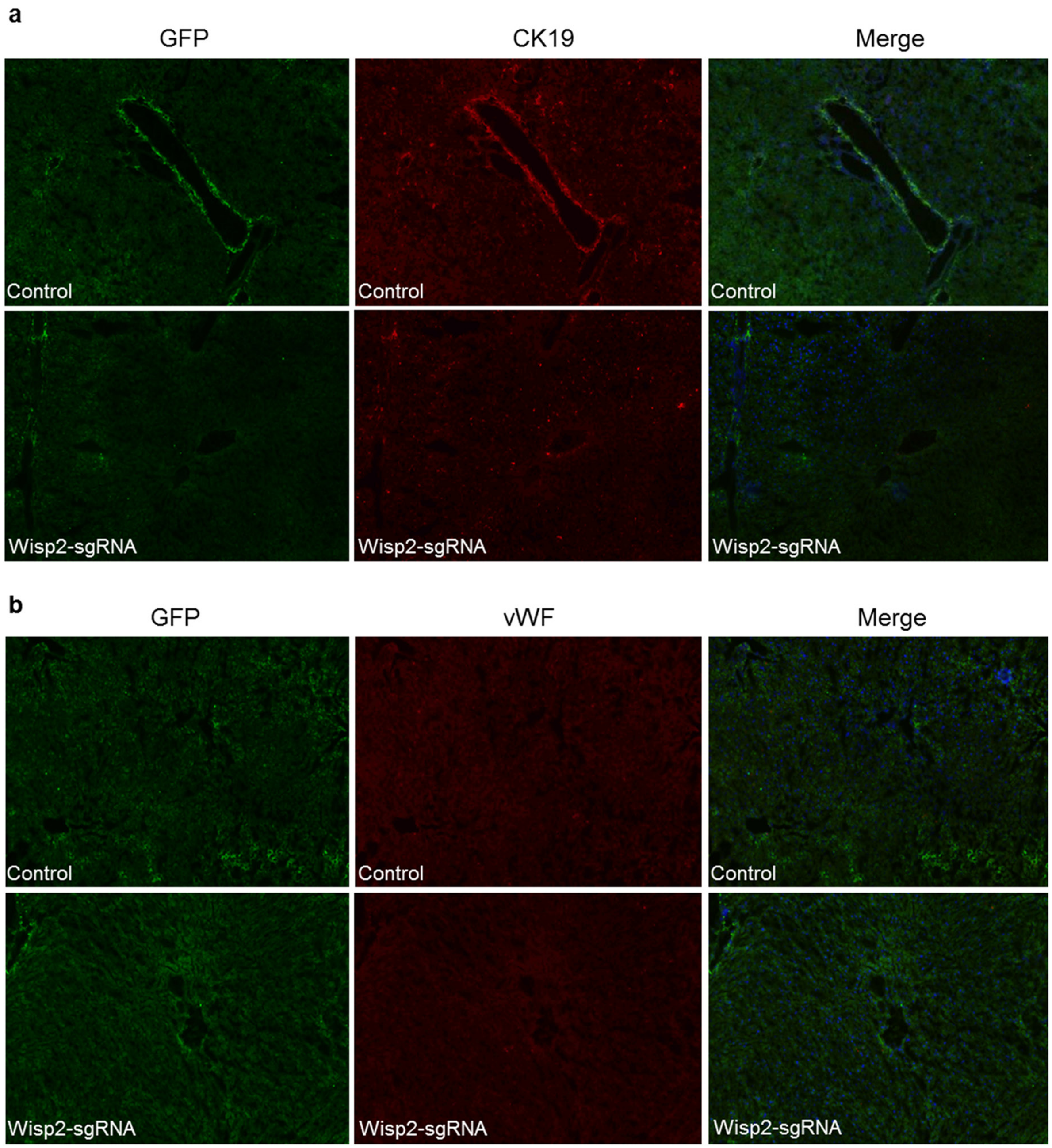

Supplementary Figure 4: Transplanted BMSCs could differentiated into bile duct cells. 18 days after BMSCs transplantation, the expression of CK19 **(a)** and vWF **(b)** were determined in liver sections. Cyrosections were stained for GFP (Green fluorescence) and CK19 or vWF (Red fluorescence). Images were merged with DAPI (Blue fluorescence) staining reveal the nuclei. Control, liver of rat transplanted with functional Wisp2 BMSCs; Wisp2-sgRNA, liver of rat transplanted with BMSCs which infected by lentivirus against Wisp2.
